# Complications of Haploidentical Hematopoietic Cell Transplantation with Post-Transplant Cyclophosphamide—A Prospective Study on Behalf of the EBMT Transplant Complications Working Party

**DOI:** 10.3390/cancers17244029

**Published:** 2025-12-18

**Authors:** Agnieszka Tomaszewska, Grzegorz W. Basak, Christophe Peczynski, Emmanuelle Polge, Pascale Ambron, William Boreland, Simona Sica, Mutlu Arat, Jakob Passweg, Jose Luis Lopez Lorenzo, Urpu Salmenniemi, Pavel Jindra, Alexander Kulagin, Rodrigo Martino Bufarull, Matthias Eder, Mohamed-Amine Bekadja, Alberto Mussetti, Charlotte E. Graham, Hélène Schoemans, Olaf Penack, Ivan Moiseev, Zinaida Perić

**Affiliations:** 1 Department of Hematology, Transplantation and Internal Medicine, Medical University of Warsaw, 02-091 Warsaw, Poland; grzegorz.basak@wum.edu.pl; 2 EBMT Paris Study Unit, Department of Haematology, Saint Antoine Hospital, INSERM UMR-S 938, Sorbonne University, 75012 Paris, France; christophe.peczynski@ebmt.org (C.P.); emmanuelle.polge@ebmt.org (E.P.); pascale.ambron@ebmt.org (P.A.); william.boreland@ebmt.org (W.B.); 3 Dipartimento di Scienze Radiologiche ed Ematologiche, Universita Cattolica S. Cuore, 00168 Rome, Italy; simona.sica@unicatt.it; 4 Department of Hematology and Transplantation, Şişli Memorial Hospital, 34384 Istanbul, Türkiye; mutlu.arat@memorial.com.tr; 5 Faculty of Medicine, Bahçeşehir University, 34734 Istanbul, Türkiye; 6 University Hospital Basel, 4031 Basel, Switzerland; jakob.passweg@usb.ch; 7 Hospital Universitario Fundación Jiménez Díaz, 28040 Madrid, Spain; jllopez@fjd.es; 8 HUS Comprehensive Cancer Center, 00029 Helsinki, Finland; urpu.salmenniemi@hus.fi; 9 Charles University Hospital, 32300 Pilsen, Czech Republic; jindra@fnplzen.cz; 10 RM Gorbacheva Research Institute, Pavlov University, 197022 St. Petersburg, Russia; kulaginad@1spbgmu.ru (A.K.); moisiv@mail.ru (I.M.); 11 Hospital Santa Creu i Sant Pau, 08025 Barcelona, Spain; rmartino@santpau.cat; 12 Department of Hematology, Hemostasis, Oncology, and Stem Cell Transplantation, Hannover Medical School, 30625 Hannover, Germany; eder.matthias@mh-hannover.de; 13 Faculty of Medicine, Ahmed Benbella-1 University, 31000 Oran, Algeria; mabekadja@yahoo.fr; 14 Institut Catalá d’Oncologia, Hospital Duran i Reynals, 199203 Barcelona, Spain; amussetti@iconcologia.net; 15 Comprehensive Cancer Centre, King’s College London, London WC2R 2LS, UK; charlotte.2.graham@kcl.ac.uk; 16 University Hospital Gasthuisberg, 3000 Leuven, Belgium; 17 Charité-CVK, University Medicine Berlin, 13353 Berlin, Germany; olaf.penack@charite.de; 18 University Hospital Centre Rijeka and School of Medicine, University of Rijeka, 51000 Rijeka, Croatia; zinaida.peric@medri.uniri.hr

**Keywords:** hematological malignancies, haploidentical hematopoietic cell transplantation, post-transplant cyclophosphamide, graft-versus-host disease, non-infectious complications, infectious complications

## Abstract

**Background:** Haploidentical hematopoietic cell transplantations (haplo-HCTs) with post-transplant cyclophosphamide (PT-Cy) are standard practice, but complications causing morbidity and mortality are not well described. **Methods:** The aim of this prospective non-interventional multicenter study was to document frequency of potential non-infectious and infection-related complications and main transplant outcomes after the first unmanipulated haplo-HCT with PT-Cy between 2017 and 2019 in 129 adult patients with hematological malignancies. The median follow-up was 37.3 months [95% CI: 34.3–39.7]. **Results:** The cumulative incidence (CI) of acute graft versus host disease (aGvHD) at day +100 was 22.4% grade II-IV [95% CI: 15.5–30.1] and 8.8% grade III-IV [95% CI: 4.6–14.6], respectively. The cumulative incidence of chronic GvHD (cGvHD) at 24 months was 25.8% [95% CI: 18.5–33.6]; extensive cGvHD was 10.9% [95% CI: 6.3–17.1], respectively. The most frequent non-infectious complications for the whole study population were mucositis—37.5% (*n* = 48); renal insufficiency—18% (*n* = 23); and cardiovascular complications—10.9% (*n* = 14). The following infection-related complications were diagnosed: bacterial in 84 (65.1%), viral in 66 (51.6%), and fungal in 24 (18.6%) recipients. Two-year OS was 58.1% [95% CI: 50.2–67.3]; NRM—27.1% [95% CI: 19.7–35]; PFS—50.4% [95% CI: 42.5–59.8]; and GRFS—38.8% [95% CI: 31.2–48.1]. About 50% of all deaths were directly caused by infection or infection-related conditions. **Conclusions:** Disease remission status at transplant significantly affected PFS, chronic GvHD, and GRFS. Although clinical applications of haplo-HCT with PTCy are widespread, the study confirms the need to reduce infection-related mortality after this type of GvHD prophylaxis.

## Simple Summary

Clinical applications of unmanipulated haploidentical hematopoietic cell transplantation with GvHD (graft versus host disease) prophylaxis using post-transplant cyclophosphamide (haplo-HCT with PTCy) have become widespread in therapy of various types of hematological malignancies. The aim of this prospective non-interventional multicenter study was to document frequency of potential non-infectious and infection-related complications and main transplant outcomes after the first haplo-HCT with PT-Cy between 2017 and 2019 in 129 adult patients. The median follow-up was 37.3 months [95% CI: 34.3–39.7]. We have identified mucositis (37.5%), renal insufficiency (18%), and cardiovascular complications (10.9%) as the three main non-infectious complications. Infections were common after haplo-HCT with PTCy—bacterial in 65.1%, viral in 51.6% and fungal in 18.6% recipients. Two-year OS was 58.1% [95% CI: 50.2–67.3]; NRM—27.1% [95% CI: 19.7–35]; PFS—50.4% [95% CI: 42.5–59.8]; and GRFS—38.8% [95% CI: 31.2–48.1]. We found that disease remission status at transplant had an impact on transplant outcomes—PFS, chronic GvHD, and GRFS.

## 1. Introduction

The use of haploidentical T-cell replete hematopoietic cell transplantations (haplo-HCT) with post-transplant cyclophosphamide (PT-Cy) is commonly applied among transplant centers, especially in patients lacking an HLA-compatible stem cell donor [1,2,3,4]. The main advantages of this procedure are the rapid access to a donor, an over 95% chance of identifying a donor, and cost-effectiveness [2,3,5]. Historically, the main complications after haplo-HCT were graft versus host disease (GvHD) and graft failure [5]. The introduction of haplo-HCT with the PT-Cy strategy has changed the landscape of post-transplant outcomes [5,6]. The cyclophosphamide is an alkylating agent metabolized by the hepatic CYP450 enzyme into an active metabolite—aldophosphamide, which is oxidized by aldehyde dehydrogenase (ALDH) [5]. In the context of haploidentical stem cell transplantation, post-transplant cyclophosphamide as a GvHD prophylaxis functionally impairs proliferating recipient and alloreactive donor T cells, which have decreased levels of ALDH, while regulatory T cells and stem cells having increased ALDH levels are spared [5,6,7]. This is the key proposed mechanism of action of PTCy in haploHCT procedures.

Despite widespread use of PT-Cy in transplantation from haploidentical donors, multicenter real-world data regarding the types and frequency of complications after these procedures are relatively small [5,6]. There are numerous retrospective reports of different non-infectious and infectious post-transplant complications occurring after haplo-HCT: cytokine release syndrome (CRS); hemorrhagic cystitis (HC); transplant-associated thrombotic microangiopathy (TA-TMA); hepatic veno-occlusive disease/sinusoidal obstruction syndrome (VOD/SOS); diffuse alveolar hemorrhage (DAH); idiopathic pneumonia syndrome (IPS); cardiac toxicity; and infections, especially of viral origin [7,8,9,10,11,12,13,14,15,16,17,18,19]. Some of them were considered probably or definitely related to the toxicity profile of cyclophosphamide [20]. The pattern of immune reconstitution, infectious complications, and immune-based complications is different after haplo transplants [5,6]. In recent retrospective reports focused on this issue, infectious complications and infection-related mortality are the major factors influencing the outcomes after haplo-HCT with PT-Cy [17,18,19,21,22,23].

In the present study, we aimed to prospectively investigate frequency of early and late non-infectious and infectious complications after PT-Cy-based haplo-HCT and determine essential survival outcomes.

## 2. Materials and Methods

### 2.1. Study Design and Inclusion Criteria

This was a prospective observational multicenter study performed on behalf of the Transplant Complications Working Party (TCWP) of the European Society for Blood and Marrow Transplantation (EBMT). The EBMT centers willing to participate were registered, and data collection followed the usual EBMT reporting guidelines (https://www.ebmt.org)—participating centers have been asked to record a MED-A D0 and MED-A D100 form (which required the minimal patient data in reporting the EBMT system ProMISE) and then to complete the Study Follow-up Form at 100 days, 12 months, and 24 months after transplantation. Data privacy was ensured according to ethical standards and the EBMT reporting guidelines. The inclusion criteria were the following: first alloHCT (previous autologous HCT was allowed), recipient age ≥ 18 years at transplantation, hematological malignancy, haploidentical donor, PT-Cy-based GvHD prophylaxis, bone marrow (BM) or peripheral blood (PB) as stem cell source, and year of transplantation between 2017 and 2019. The only exclusion criterion was ex vivo T-cell depletion.

In the present study, we aimed to prospectively investigate frequency of main early and late non-infectious and infectious complications after PT-Cy-based haplo-HCT and determine essential survival outcomes. We have performed analyses in the whole study population and according to disease remission status at transplant. The rationale for analyses in the CR (complete remission) versus the no-CR group was expected unfavorable results and outcomes in patients transplanted in an active disease. According to the study protocol, the following non-infectious complications were collected: mucositis, renal insufficiency, cardiovascular complications, VOD/SOS, TA-TMA, DAH, ELS (endothelial leakage syndrome), ES (engraftment syndrome), non-infective HC (haemorrhagic cystitis), PRES (posterior reversible encephalopathy syndrome), IPS (idiopathic pneumonia syndrome), BOS (bronchiolitis obliterans syndrome), COP (cryptogenic organizing pneumonia), osteoporosis, endocrine effects, PTLD (post-transplant lymphoproliferative disease) and other secondary malignancies, early graft loss, and graft failure and graft versus host disease (GvHD). Among infectious complications, data on bacterial, viral, and fungal infections and their main clinical presentation were collected. Data for all non-infectious and infectious complications were not collected, so only frequencies are presented and not incidences. The main recipient, donor, and transplant procedure characteristics, such as age and gender, cytomegalovirus (CMV) serostatus, disease diagnosis and its status at transplantation, HCT-CI (hematopoietic cell transplantation—comorbidity index), Karnofsky performance status, intensity of conditioning regimen, stem cell source, and GvHD prophylaxis regimen, were included. The definition of conditioning intensity, grading of acute GvHD and chronic GvHD, early graft loss, and graft failure were performed using published criteria [7,24,25,26,27].

The list of transplant centers contributing data to this study and the list of collected parameters are presented in the Supplementary Materials.

### 2.2. Statistical Methods

The primary endpoint was the frequency of each non-infectious and infectious complication after transplant. Secondary endpoints comprised survival outcomes: overall survival (OS), progression-free survival (PFS), relapse incidence (RI), non-relapse mortality (NRM), GvHD-relapse-free survival (GRFS), acute GvHD (aGvHD) grades II–IV and III–IV, and overall and extensive chronic GvHD (cGvHD).

The median values and ranges were used for continuous variables and percentages for categorical variables. Patient-, disease-, and transplant-related variables were compared between the two groups (CR at transplant vs. no CR) using the Mann–Whitney *U* test for numerical variables, and the Chi-squared or Fisher’s exact test for categorical variables.

OS was defined as the time from alloHCT to death, regardless of the cause. PFS was defined as the time from alloHCT to relapse or death from any cause. RI was defined as disease recurrence after alloHCT. NRM was defined as death without previous relapse. GRFS was defined as the first occurrence of grade III-IV acute GvHD or extensive chronic GvHD or relapse/progression or death from any cause. Acute GvHD was graded according to the modified Seattle-Glucksberg criteria and chronic GvHD according to the revised Seattle criteria. Neutrophil engraftment was defined as an achievement of an absolute neutrophil count (ANC) ≥ 0.5 G/L for 3 days without growth factor support, and platelet engraftment as a platelet count (PLT) > 50 G/L without transfusion. All the outcomes were measured from the time of stem cell infusion. The probabilities of OS, PFS, and GRFS were calculated with the Kaplan–Meier test, and those of RI, NRM, and acute and chronic GvHD with the cumulative incidence estimator to accommodate for competing risks. For NRM, relapse was the competing risk, and for relapse, the competing risk was NRM. For acute and chronic GvHD, death without the event and relapse were the competing risks. Median follow-up time was estimated using the reverse Kaplan–Meier method. Univariate analyses were performed using the log-rank test for OS and PFS, while Gray’s test was used for competing risk outcomes.

Statistical analyses were performed with R 4.1.2 (R Core Team (2021). R: A language and environment for statistical computing. R Foundation for Statistical Computing, Vienna, Austria. URL https://www.R-project.org/).

## 3. Results

### 3.1. Patients, Disease, and Transplant Characteristics

A total of 129 adult patients undergoing haplo-HCT using PT-Cy between 2017 and 2019 were included. The analysis was performed for the whole study population and compared two groups of recipients: those in CR (complete remission) (*n* = 68) at transplant versus no-CR disease status (*n* = 58).

The patient, donor, and procedure characteristics for the whole study population and according to remission status at haplo-HCT are shown in Table 1.

Patients in the no-CR group were older (median age 58.9 vs. 45.1; *p* < 0.001), and 75.8% of them were diagnosed with lymphoma and myelodysplastic/myeloproliferative disorders, while 86.8% of the CR group patients were diagnosed with acute leukemias (*p* < 0.001). Bone marrow was the main stem cell source in the no-CR group (69% vs. 48.5% in the CR group; *p* = 0.021).

The median follow-up was 37.3 months [95% CI: 34.3–39.7]. The two main causes of death were the original disease and infection. The original disease was diagnosed as the main cause of death in 34.6% of patients in the CR group and in 36.7% in the no-CR group, respectively. Infection-related deaths were reported in 26.9% of the CR patients and in 46.7% of the no-CR recipients, respectively.

### 3.2. Non-Infectious Complications

As mentioned above, we analyzed occurrence of all significant non-infectious post-transplant complications, including endothelial syndromes, organ toxicities, GvHD, and graft failure.

**Figure 1:**
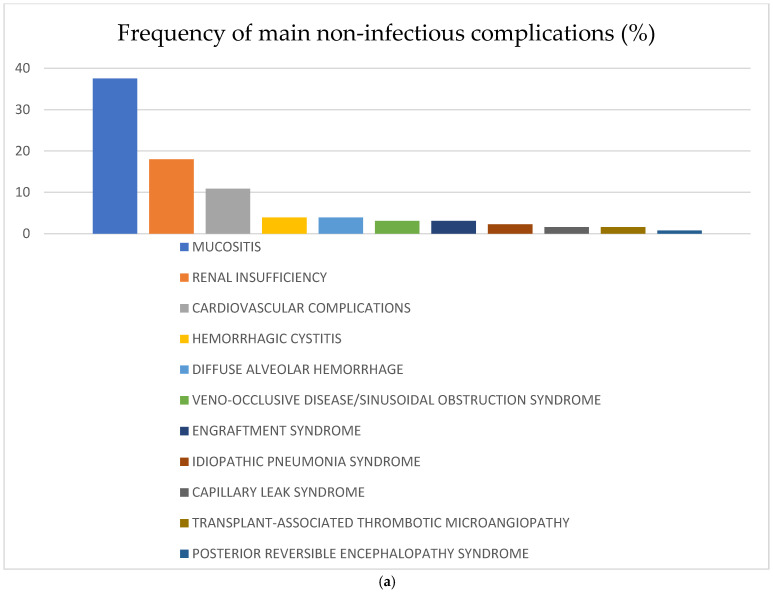

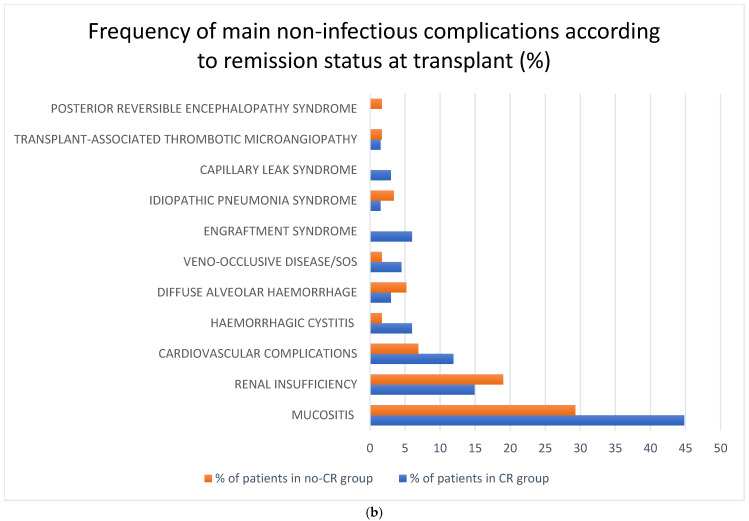
(**a**) Frequency of non-infectious complications after haplo-HCT with PT-Cy in the whole population; (**b**) frequency of non-infectious complications after haplo-HCT with PT-Cy according to remission status at transplant. Abbreviations: SOS—sinusoidal obstruction syndrome.

The most frequent non-infectious complications for the whole study population were mucositis—37.5% (*n* = 48); renal insufficiency—18% (*n* = 23); and cardiovascular —10.9% (*n* = 14). The rest of the analyzed non-infectious complications were diagnosed in less than 5% of the recipients: HC—3.9% (*n* = 5), DAH—3.9% (*n* = 5), VOD/SOS—3.1% (*n* = 4), engraftment syndrome—3.1% (*n* = 4), thrombotic microangiopathy—1.6% (*n* = 2), capillary leak syndrome (CLS)—1.6% (*n* = 2), idiopathic pneumonia syndrome—2.3% (*n* = 3), posterior reversible encephalopathy syndrome—0.8% (*n* = 1) and osteoporosis—3.1% (*n* = 4). There were no reported events of endocrine complications, post-transplant lymphoproliferative disease (PTLD), or other secondary malignancies. Some of the complications mentioned above were observed more often in the CR vs. no-CR group: mucositis 44.8% vs. 29.3%, cardiovascular complications 11.9% vs. 6.9%, HC 6% vs. 1.7%, VOD/SOS 4.5% vs. 1.7%, ES 6% vs. 0%, and CLS 3% vs. 0%, respectively. Renal complications were diagnosed most frequently in the no-CR vs. CR group: 19% vs. 14.9%, respectively. Frequency of the main non-infectious complications in the whole study population and according to remission status is shown in Figure 1.

Cytokine release syndrome (CRS) was diagnosed in eight (6.2%) patients, mostly in grade 1 according to Lee criteria (only one patient developed CRS of grade 2), and six in the CR group [27]. It should be mentioned that according to certain transplant center protocols, steroids were used in 14 (10.9%) and calcineurin inhibitors in 16 (12.4%) recipients before PT-Cy administration. There was no report of tocilizumab administration in the analyzed population.

Neutrophil engraftment (ANC—absolute neutrophil count ≥ 0.5 G/L) occurred in 109 (94.8%) patients, and platelets (>50 G/L) in 79 (63.2%). Growth factors were used in 97 (75.8%) recipients, mostly G-CSF (in 94 patients). Full donor chimerism was achieved in 100 (90.1%) patients.

Early graft loss was diagnosed in five patients: two in the CR group and three in the no-CR group, respectively. Graft failure was diagnosed in six (4.7%) patients: one in the CR group and five in the no-CR group.

### 3.3. GvHD

**Figure 2:**
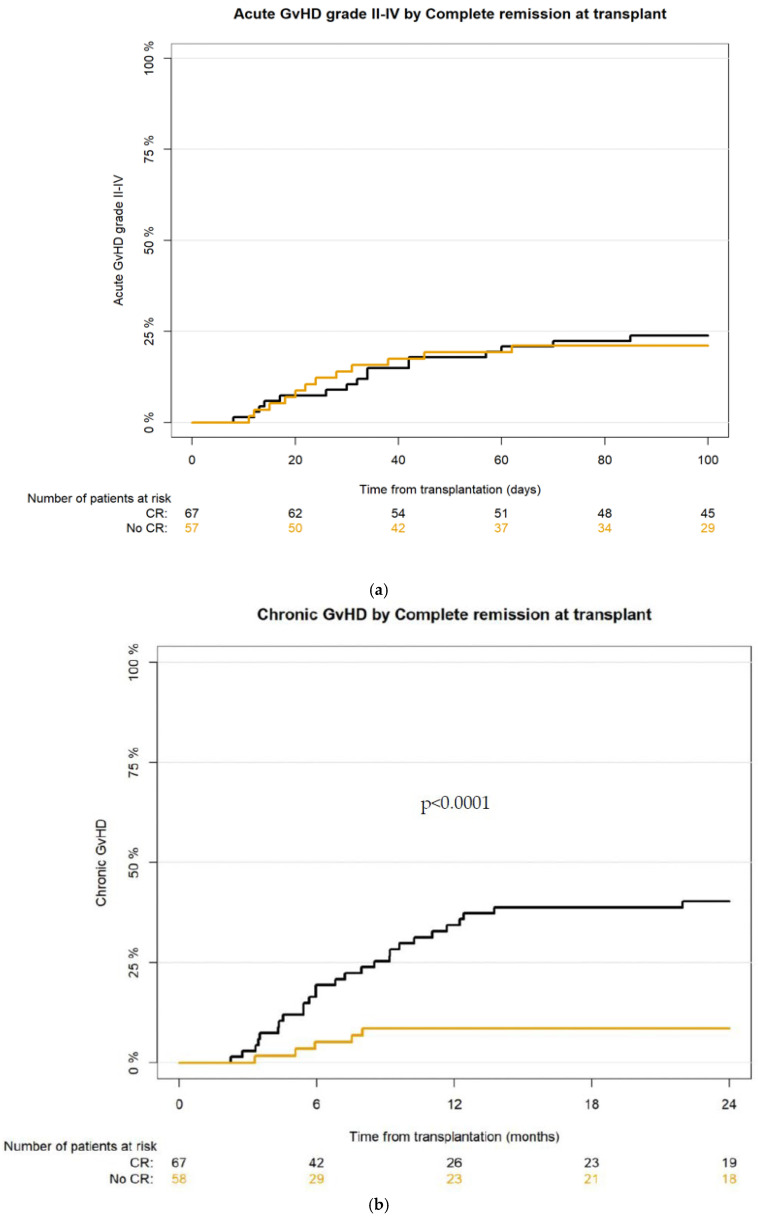
(**a**) Cumulative incidence of aGvHD grade II-IV after haplo-HCT with PT-Cy in CR vs. no-CR group; (**b**) cumulative incidence of cGvHD after haplo-HCT with PT-Cy in CR vs. no-CR group.

The cumulative incidence of acute GvHD at day +30 and at day +100 for grade II-IV acute GvHD was 12% [95% CI: 7–18.4] and 22.4% [95% CI: 15.5–30.1], and for grade III-IV aGvHD 5.6% [95% CI: 2.5–10.6] and 8.8% [95% CI: 4.6–14.6], respectively. There was no significant difference between the CR vs. no-CR group in aGvHD incidence—for grade II-IV at day +30 10.4% [95% CI: 4.6–19.2] vs. 14.8% [95% CI: 6.9–25.6] and at day +100 23.9% [95% CI: 14.4–34.7] vs. 22.2% [95% CI: 12.2–34.1], respectively (Figure 2a). Complete resolution of aGvHD was achieved in 75.7% of patients; most of them (83.3%) were treated with corticosteroids.

The cumulative incidence for cGvHD at 12 months and at 24 months was 21.9% [95% CI: 15.1–29.4] and 25.8% [95% CI: 18.5–33.6], respectively; for extensive cGvHD—9.4% [95% CI: 5.1–15.2] and 10.9% [95% CI: 6.3–17.1], respectively. According to the National Institute of Health (NIH) scoring severity system [26], mild cGvHD was recognized in 13 (39.4%), moderate in 15 (45.5%), and severe in 5 (15.2%) recipients who developed chronic GvHD.

The incidence of cGvHD and extensive cGvHD was significantly higher in the CR group of patients, CI for cGvHD at 12 months for CR group was 34.3% [95%CI: 23.2–45.8] vs. 8.6% [95% CI: 3.1–17.7] in the no-CR group, and at 24 months 40.3% [95% CI: 28.4–51.9] vs. 8.6% [95% CI: 3.1–7.7], respectively (*p* < 0.001) (Figure 2b). The incidence of extensive cGvHD at 12 months for CR group was 13.4% [95% CI: 6.6–22.8] vs. 5.2% [95% CI: 1.4–13.8] in the no-CR group, and at 24 months 16.4% [95% CI: 8.7–26.3] vs. 5.2% [95% CI: 1.3–13.1], respectively.

### 3.4. Infectious Complications

**Figure 3:**
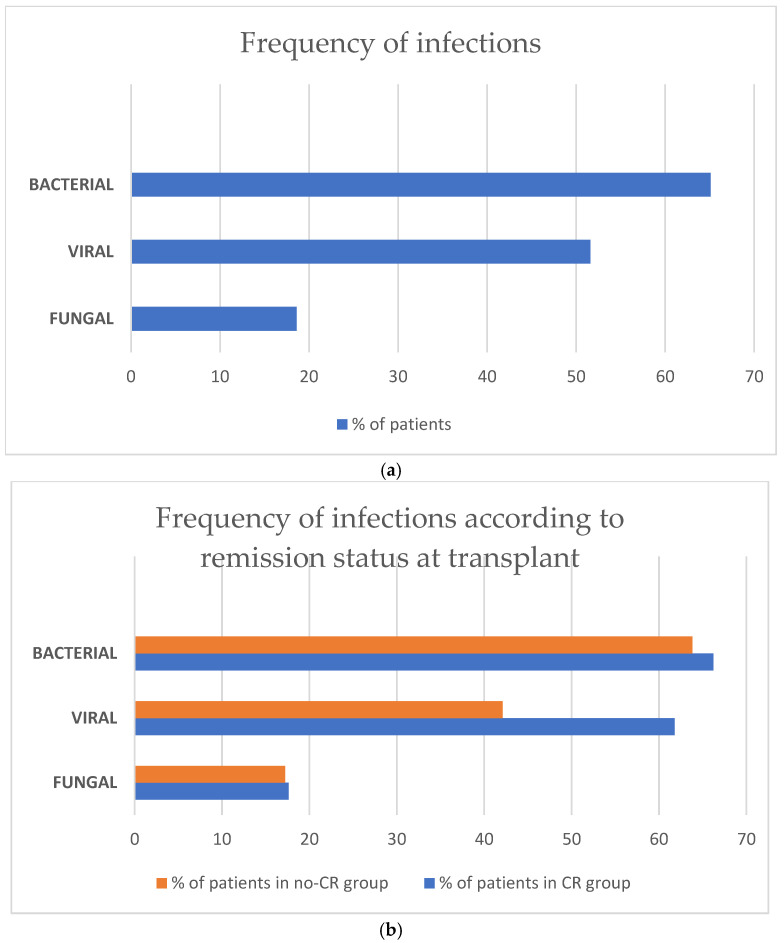
(**a**) Frequency of infectious complications after haplo-HCT with PT-Cy in the whole population. (**b**) Frequency of infectious complications after haplo-HCT with PT-Cy according to remission status at transplant.

Infection-related complications were frequently diagnosed in the whole study population: bacterial in 84 (65.1%), viral in 66 (51.6%), and fungal in 24 (18.6%) (Figure 3). There was only one case of infection (0.8%) of parasitic origin. There were no clinically relevant differences according to frequency of bacterial and fungal infectious complications between the CR vs. the no-CR group: bacterial infection was diagnosed in 66.2% in the CR and 63.8% in the no-CR group, and fungal—in 17.6% and 17.2%, respectively. A disparity was observed in viral infections—61.8% in the CR vs. 42.1% in the no-CR recipients, respectively. The most frequent clinical presentations of infection were pneumonia in 42 (32.6%), septic shock in 20 (15.5%), haemorrhagic cystitis in 20 (15.5%), gut infection in 19 (14.7%), skin infection in 9 (7%), and central nervous system involvement in 4 (3.1%) patients, respectively.

Acute respiratory distress syndrome (ARDS) due to infection was diagnosed in 14 (10.9%) patients, and multi-organ failure (MOF) in 12 (9.3%) of them. Neither infectious-related hepatitis nor nephritis was present in the whole population.

### 3.5. Transplantation Outcomes

The analyzed transplantation outcomes are presented in Table 2.

**Figure 4:**
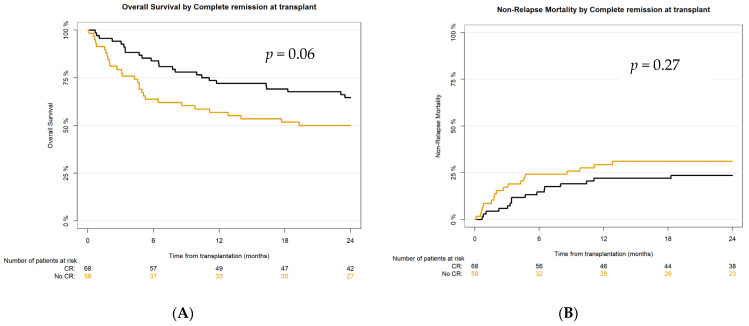

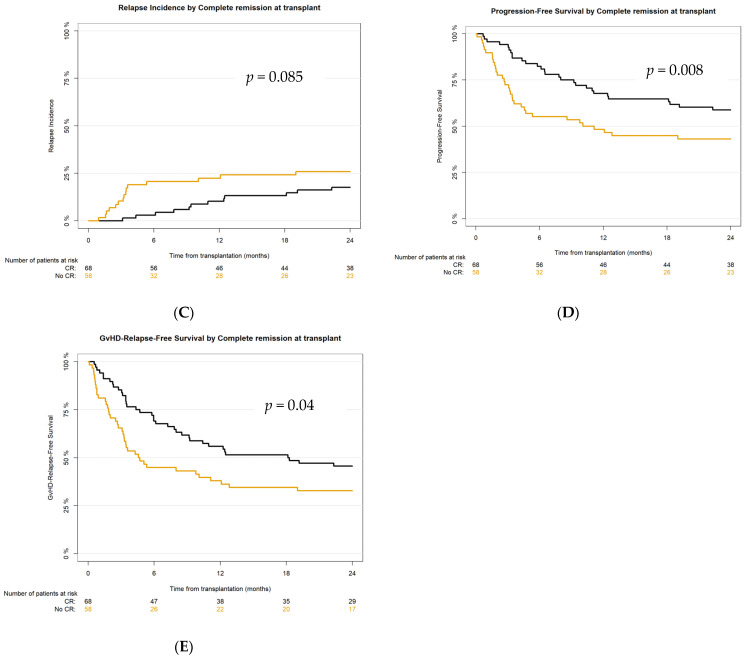
Survival outcomes after haplo-HCT with PT-Cy according to remission status at transplant: (**A**) overall survival (OS), (**B**) non-relapse mortality (NRM), (**C**) relapse incidence (RI), (**D**) progression-free survival (PFS), (**E**) GvHD-relapse-free survival (GRFS).

Main survival outcomes according to remission status at transplant are presented in Figure 4.

## 4. Discussion

The present study prospectively and comprehensively analyzed real-world multicenter data on non-infectious and infection-related complications and main transplant outcomes after unmanipulated haplo-HCT with PT-Cy in 129 adult patients with hematological malignancies.

For non-infectious complications, we focused mainly on organ toxicities, complications of endothelial origin, and graft versus host disease. Among organ toxicities, besides mucositis, the most frequent complication was renal insufficiency, diagnosed in 18% (*n* = 23) of patients. Renal insufficiency was lower in our cohort than published data in the post-allogeneic stem cell transplant setting, which reports a frequency of 50% [28,29]. The main limitation of our analysis is lack of data on severity grading of adverse events.

Cardiovascular complications were the next most frequently occurring organ toxicity, recognized in 10.9% (*n* = 14) of recipients. Cardiac complications after haplo-HCT with PT-Cy have been investigated recently in retrospective studies showing the cumulative incidence as high as 19% and it was settled that they are dose-dependent [15,30]. Our prospective and real-life data show lower frequency of cardiac toxicity, and it is worth highlighting that over 99% of patients received cyclophosphamide in a standard total dose of 100 mg/kg, 55.8% of them in +3 and +4 days, and 42.6% according to the Genova protocol, i.e., +3 and +5 days after transplant [31]. Recently, data was published on cardiac complications after haplo-HCT with PT-Cy in 58 elderly patients (aged ≥ 65 years) and those with cardiac comorbidities [32]. Authors compared the standard cyclophosphamide dose of 100 mg/kg in 25 recipients with a reduced total dose to 70 mg/kg combined with a low dose of antithymocyte globulin in 33 [32]. Cardiac complications were reported in 44% of patients with standard dose versus 12% in the reduced dose group, but it was still a higher proportion than in our cohort of 129 recipients. For older patients and those with cardiovascular diseases, this reduced dose strategy could be a valid option in mitigating cardiac toxicity after haplo-HCT with PT-Cy [32].

According to our findings, frequency of the main complications of endothelial origin was less than 4% each. These results are lower than published data [7]. For example, SOS/VOD in our study was diagnosed in only four recipients (3.9%), while incidence of this complication in adults is diagnosed in about 5–10% depending on the risk group [7,14,33,34]. One of the possible explanations is a wider time gap between conditioning and administration of calcineurin inhibitors compared to conventional protocols. The other non-infectious complication that is expected, due to high doses of cyclophosphamide, is haemorrhagic cystitis (HC) [7,8,9,10,11]. An early HC after haploPTCy was reported in almost 30% of patients [10], but the main cause of HC in haplo transplants is viral infections—HC in general was diagnosed in 55–62% of recipients with BK PyV (BK Polyomavirus) as a main factor in 91% of them [9,10,11]. In our cohort, non-infectious HC was a rare complication recorded in 5 (3.9%) patients, and HC of infectious origin in 20 (15.5%) of them, which constitutes a lower frequency than in published data [7,8,9,10,11].

Cytokine release syndrome (CRS) was reported as a common complication in haploPTCy transplant recipients, especially with onset after day 0 and before cyclophosphamide infusion, in more than 70% of patients [16,35,36,37]. In our study, we confirmed CRS only in 8 (6.2%) patients, and with severity mostly of grade 1. An important information to note is that steroids were used in 14 (10.9%) and calcineurin inhibitors in 16 (12.4%) recipients before PT-Cy administration, according to each center’s protocol. It is probable that this complication was underdiagnosed in the analyzed population due to the absence of clear criteria, which were refined after the initiation of the study [38].

In addition, almost 95% of the patients in this study engrafted, and graft failure was rare—we identified this complication in 6 (4.7%) patients. In published data, graft failure or poor graft function after haplo transplants, even with the use of PT-Cy, is still reported as an important issue with frequency reaching 27.5% [7,39].

Regarding aGvHD, the incidence of this complication in our study was similar to that reported recently after haplo transplants with PT-Cy and significantly lower than in historically published data reaching 40% [1,7,35,40]. Cumulative incidence of acute GvHD grade II-IV at day +100 was 22.4% and grade III-IV 8.8% compared with 18–33% and 9.4–13%, respectively, reported by others [41,42,43]. The rate of any grade chronic GvHD was 25.8% and is definitely lower than 46.9% as previously published, and in our cohort, extensive GvHD was 10.9% at 2 years, which is close to results reported by others with a frequency of 15.6% and 3.1%, respectively [7,41,42,43].

Infections are considered the main complication of haplo-HCT with PT-Cy, affecting NRM, and in our cohort, they were the main or contributing cause of death, as previously described in other studies [17,18,21,35]. The frequency of bacterial infections was the highest in the analyzed population, followed by viral and, less commonly, fungal infections, but in the CR group, we noticed the predominance of viral infections. In published reports, there are differences in cumulative incidence of infection by pathogen type—in some of them, viral, especially due to CMV, are the most often diagnosed infections, but bacterial in others [17,18,21,22,23,31,44,45,46,47].

The main transplant outcomes in our study were not inferior to what was previously reported [7,41,42,48,49,50]. According to NRM, the 2-year CI in the analyzed population was 27.1% in comparison with the published 31% [39], and there was no significant difference between the CR vs. the no-CR group. OS in our cohort at 2 years was 58.1% (better in the CR group—64.6%)—in previously reported studies it was 54–57% [7,41,42,48,49,50]. PFS at 24 months for the whole study population was 50.4% (for the CR group 58.8%)—in published reports, 49–54% [7,41,42,50]. And finally, 2-year GvHD-relapse-free survival in the analyzed cohort was 38.8%, in the CR group—45.6%, as compared to about 36% in previous reports [41,51]. However, active disease was diagnosed in almost 45% (*n* = 58) of recipients at transplant, and even in this very poor prognosis cohort, haplo-HCT with PT-Cy was feasible and effective, resulting in 2-year OS, PFS, and GRFS at 50%, 43.1%, and 32.8%, respectively.

## 5. Conclusions

In conclusion, our prospective study provided multicenter real-world evidence on non-infectious and infection-related complications of T cell-replete haploidentical stem cell transplantation with post-transplant cyclophosphamide and emphasizes the impact of infections on transplant outcomes. For 28 patients of all 56 reported deaths in the study, the mortality was directly caused by infections or was infection-related. Non-infectious complications were, in general, less common in our cohort than what has been previously described in haplo-HCT with PT-Cy. Disease remission status at transplant significantly affected PFS, chronic GvHD, and GRFS. Refinement of the transplantation procedure with PT-Cy should be the subject of further studies.

## Figures and Tables

**Table 1:** Patient, donor, and procedure characteristics for the whole study population and according to remission status at haplo-HCT.

Variables	All Patients N = 129 (%)	CR Group * N = 68 (%)	No-CR Group * N = 58 (%)	*p*
Median age, y, min-max, [IQR]	52.8 (18–72.9) [34.3–62.5]	45.1 (18–72.5) [30.7–56.8]	58.9 (18.8–72.9) [47.1–64.6]	0.0002
Donor age, y, min-max, [IQR]	36.4 (13.6–66.3) [27.7–45.4]	39 (17.6–61.7) [29.3–48.3]	33.2 (13.6–66.3) [24.7–42.5]	0.011
Patient sex				
Male	79 (61.2%)	42 (61.8%)	36 (62.1%)	0.97
Female	50 (38.8%)	26 (38.2%)	22 (37.9%)	
Donor sex				
Male	89 (69%)	49 (72.1%)	39 (67.2%)	0.56
Female	40 (31%)	19 (27.9%)	19 (32.8%)	
Patient CMV				
Negative	20 (15.7%)	10 (14.7%)	10 (17.2%)	0.7
Positive	107 (84.3%)	58 (85.3%)	48 (82.8%)	
Donor CMV				
Negative	34 (26.8%)	14 (20.9%)	19 (33.3%)	0.12
Positive	93 (73.2%)	53 (79.1%)	38 (66.7%)	
Donor/patient CMV serostatus				
+/+	82 (65.6%)			
+/−	10 (8.0%)			
−/−	10 (8.0%)			
−/+	23 (18.4%)			
Missing	4			
Diagnosis				<0.0001
Acute leukemia	73 (56.6%)	59 (86.8%)	14 (24.1%)	
Lymphoma	29 (22.5%)	6 (8.8%)	22 (37.9%)	
Myelodysplastic/ Myeloproliferative neoplasm	27 (20.9%)	3 (4.4%)	22 (37.9%)	
Graft source				
BM	74 (57.4%)	33 (48.5%)	40 (69.0%)	0.021
PB	55 (42.6%)	35 (51.5%)	18 (31.0%)	
Previous autologous transplantation				
No	112 (86.8%)	61 (89.7%)	48 (82.8%)	0.26
Yes	17 (13.2%)	7 (10.3%)	10 (17.2%)	
HCT-CI				
0	49 (39.5%)	27 (40.9%)	22 (38.6%)	0.84
1–2	33 (26.6%)	18 (27.3%)	14 (24.6%)	
≥3	42 (33.9%)	21 (31.8%)	21 (36.8%)	
Missing	5	2	1	
KPS				
≥90	46 (36.5%)	28 (42.4%)	17 (29.3%)	0.13
<90	80 (63.5%)	38 (57.6%)	41 (70.7%)	
Missing	3	2	0	
Intensity of conditioning				
MAC	94 (72.9%)	51 (75%)	42 (72.4%)	0.74
RIC	35 (27.1%)	17 (25%)	16 (27.6%)	
Conditioning regimen				
Bu-based	88 (68.2%)	45 (66.2%)	41 (70.7%)	
Treo-based	4 (3.1%)	4 (5.9%)	0 (0%)	
Mel-based	9 (7.0%)	2 (2.9%)	6 (10.3%)	
TBI-based	27 (20.9%)	16 (23.5%)	11 (19%)	
Other	1 (0.8%)	1 (1.5%)	0 (0%)	
GvHD prophylaxis				
CsA + MMF + PT-Cy	91 (70.5%)	46 (67.6%)	44 (75.9%)	
TACRO + MMF + PT-Cy	19 (14.7%)	10 (14.7%)	8 (13.8%)	
CsA + TACRO + MMF + PT-Cy	6 (4.7%)	3 (4.4%)	3 (5.2%)	
TACRO + SIRO + MMF + PT-Cy	5 (3.9%)	4 (5.9%)	0 (0%)	
CsA + MTX + MMF + PT-Cy	3 (2.3%)	3 (4.4%)	0 (0%)	
Other	5 (3.9%)	2 (3%)	2 (3.6%)	
PT-Cy daily dose (mg/kg m.c.)				
50	127 (99.2%)	68 (100%)	56 (96.6%)	
40	1 (0.8%)	0 (0%)	1 (1.7%)	
Missing	1	0 (0%)	1 (1.7%)	
Days of PT-Cy administration				
+3 and +4	72 (55.8%)	36 (52.9%)	33 (56.9%)	
+3 and +5	55 (42.6%)	32 (47.1%)	23 (39.7%)	
+2 and +3	1 (0.8%)	0 (0%)	1 (1.7%)	
Only +3	1 (0.8%)	0 (0%)	1 (1.7%)	

Abbreviations: CR—complete remission; CMV—cytomegalovirus; BM—bone marrow; PB—peripheral blood; HCT-CI—hematopoietic cell transplantation-specific comorbidity index; KPS—Karnofsky performance status; MAC—myeloablative conditioning; RIC—reduced intensity conditioning; Bu—busulfan; Treo—treosulfan; Mel—melphalan; TBI—total body irradiation; CsA—cyclosporine A; MMF—mycophenolate mofetil; TACRO—tacrolimus; SIRO—sirolimus; MTX—methotrexate. * For 3 patients, data regarding disease remission status were missing.

**Table 2:** Univariate analysis of transplantation outcomes for the whole study population and according to remission status at haplo-HCT.

Outcome (at 24 Months)	% [95% CI] for Whole Study Population	% [95% CI] for CR Group	% [95% CI] for no-CR Group	*p*
OS	58.1% [50.2–67.3]	64.6% [54.2–77.1]	50% [38.7–64.7]	0.06
PFS	50.4% [42.5–59.8]	58.8% [48.2–71.8]	43.1% [32.1–57.9]	0.008
NRM	27.1% [19.7–35]	23.5% [14.2–34.2]	31% [19.6–43.2]	0.27
RI	22.5% [15.7–30]	17.6% [9.6–27.6]	25.9% [15.3–37.7]	0.085
GRFS	38.8% [31.2–48.1]	45.6% [35.2–59.1]	32.8% [22.7–47.4]	0.04

Abbreviations: OS—overall survival; PFS—progression-free survival; NRM—non-relapse mortality; RI—relapse incidence; GRFS—GvHD-relapse-free survival.

## Data Availability

The datasets used and analyzed during the current study are available from the corresponding author on reasonable request.

## Supplementary Materials

The following supporting information can be downloaded at https://www.mdpi.com/article/10.3390/cancers17244029/s1. Table S1: The list of transplant centers contributing data to this study. Table S2: The list of main collected parameters. Infection-related complications. Table S3: The list of main collected parameters. Non-infectious complications.
