# Supplementary material for: Complications of Haploidentical Hematopoietic Cell Transplantation with Post-Transplant Cyclophosphamide—A Prospective Study on Behalf of the EBMT Transplant Complications Working Party
Source: Cancers (Basel). 2025 Dec 18;17(24):4029. doi: 10.3390/cancers17244029 (PMC12731586; doi:10.3390/cancers17244029)
Supplement: Supplementary file 1 [file cancers-17-04029-s001.zip › cancers-4049599-supplementary.pdf]

Table S1. The list of transplant centers contributing data to this study:

Centre	Number of patients
Rome, Universita Cattolica S. Cuore	32
Istanbul, Şişli Memorial Hospital, Department of Hematology and Transplantation	31
Basel, University Hospital Basel	22
Madrid, Hospital Universitario Fundación Jiménez Díaz	14
Helsinki, HUS Comprehensive Cancer Center	12
Pilsen, Charles University Hospital,	9
St.Petersburg, RM Gorbacheva Research Institute, Pavlov University,	5
Barcelona, Hospital Santa Creu i Sant Pau	2
Hannover, Hannover Medical School	1
Oran, Ahmed Benbella-1 University, Faculty of Medicine	1

Table S2. The list of main collected parameters. Infection-related complications.

Variable	Level	n=129
**Bacteria**	No	45 (34.9%)
	Yes	84 (65.1%)
**Fungi**	No	105 (81.4%)
	Yes	24 (18.6%)
**Viruses**	No	62 (48.4%)
	Yes	66 (51.6%)
	missing	1
**Parasites**	No	128 (99.2%)
	Yes	1 (0.8%)
**Septic shock**	No	109 (84.5%)
	Yes	20 (15.5%)
**Acute Respiratort Distress Syndrome (ARDS)**	No	115 (89.1%)
	Yes	14 (10.9%)
**Respiratory Invasive Fungal disease**	No	124 (96.9%)
	Yes	4 (3.1%)
	missing	1
**Multi organ Failure due to infection**	No	117 (90.7%)
	Yes	12 (9.3%)
**Pneumonia**	No	87 (67.4%)
	Yes	42 (32.6%)
**Hepatitis**	No	129 (100%)
**CNS infection**	No	124 (96.9%)
	Yes	4 (3.1%)
	missing	1
**Gut infection**	No	110 (85.3%)
	Yes	19 (14.7%)
**Skin infection**	No	120 (93%)

	Yes	9 (7%)
**Hemmorhagic cystitis (infective)**	No	109 (84.5%)
	Yes	20 (15.5%)
**Renitis**	No	129 (100%)
**Other**	No	106 (82.2%)
	Yes	23 (17.8%)

Table S3. The list of main collected parameters. Non-infectious complications.

**Variable**	**Level**	**n=129**
**Graft failure**	No	122 (95.3%)
	Yes	6 (4.7%)
	missing	1
**Idiopathic pneumonia syndrome**	No	125 (97.7%)
	Yes	3 (2.3%)
	missing	1
**Haemorrhagic cystitis (noninfective)**	No	123 (96.1%)
	Yes	5 (3.9%)
	missing	1
**Diffuse alveolar haemorrhage**	No	123 (96.1%)
	Yes	5 (3.9%)
	missing	1
**Engraftment syndrome**	No	124 (96.9%)
	Yes	4 (3.1%)
	missing	1
**Endothelial leakage syndrome**	No	125 (98.4%)
	Yes	2 (1.6%)
	missing	2
**Veno-occlusive disease (VOD/SOS)**	No	124 (96.9%)
	Yes	4 (3.1%)
	missing	1
**Thrombotic microangiopathy (TMA)**	No	126 (98.4%)
	Yes	2 (1.6%)
	missing	1
**Posterior reversible encephalopathy syndrome (PRES)**	No	127 (99.2%)
	Yes	1 (0.8%)
	missing	1
**Mucositis**	No	80 (62.5%)
	Yes	48 (37.5%)
	missing	1

**Bronchiolitis obliterans syndrome (BOS)**	No	128 (100%)
	missing	1
**Cryptogenic organizing pneumonia (BOOP)**	No	128 (100%)
	missing	1
**Osteoporosis**	No	124 (96.9%)
	Yes	4 (3.1%)
	missing	1
**Endocrine complications**	No	127 (99.2%)
	Yes	1 (0.8%)
	missing	1
**Renal insufficiency**	No	105 (82%)
	Yes	23 (18%)
	missing	1
**Cardiovascular complications**	No	114 (89.1%)
	Yes	14 (10.9%)
	missing	1
**Secondary malignancies**	No	128 (100%)
	missing	1
**Post-transplant lymphoproliferative disease**	No	128 (100%)
	missing	1
